# Tr14 gel compared to diclofenac gel after acute unilateral ankle sprain: an Individual Patient Data (IPD) meta-analysis of two multi-center trials

**DOI:** 10.1186/s12891-026-09802-0

**Published:** 2026-05-01

**Authors:** J. Vester, L. Gerdesmeyer, G. Kerkhoffs

**Affiliations:** 1 idv Data Analysis & Study Planning, Gauting, Germany; 2 Klinik für Orthopädie und Unfallchirurgie, Universitätsklinikum Schleswig-Holstein, Kiel, Germany; 3 Department of Orthopaedic Surgery and Sports Medicine, Amsterdam Movement Sciences, Amsterdam University Medical Centers, University of Amsterdam, Amsterdam, the Netherlands

**Keywords:** Traumeel, Tr14, TRAUMED, TAASS, RCT, Lateral Ankle Sprain, Inflammation, Re-injury, Musculoskeletal Pain, Inflammation Resolution, Pro-resolution

## Abstract

**Background:**

Traumeel (Tr14) and diclofenac gels are commonly prescribed topical treatments for Grade I-II acute lateral ankle sprain (LAS). A meta-analysis of two multi-center trials was performed to compare their clinical efficacy and supplement the evidence in support of topical treatments.

**Methods:**

Data from two Phase III prospective, randomised, double-blind, reference-controlled clinical trials, TAASS (2013) and TRAUMED (2024) were examined. An Individual Patient Data (IPD) meta-analysis was performed on those with an initial pain VAS (Visual Analogue Scale) score ≥ 50 mm, using the statistical analysis plan from the latter to ensure methodological consistency.

**Results:**

A total of 628 patients underwent meta-analysis (mean baseline pain VAS = 72.3, mean age = 32.5). The primary endpoint of the IPD meta-analysis, Area Under the Curve (AUC) of pain VAS scores, indicated beneficial effects of Tr14 gel compared with diclofenac gel, statistically significant on Days 4, 7, and 14 (Day 4 mean difference MD_AUC-META_ = -6.9 [95%CI -13.7 to -0.1], P_AUC-META_ = 0.05; Day 7 MD_AUC-META_ = -26.3 [95%CI -40.4 to -12.2], P_AUC-META_ = 0.0003; Day 14 MD_AUC-META_ = -63.6 [95%CI -92.3 to -34.8]), P_AUC-META_ < 0.0001). A robust Wilcoxon-Mann-Whitney (WMW) analysis revealed percent changes from baseline in pain and function that were significantly superior for Tr14 gel in the pre-planned method of synthesis, compared to diclofenac gel, on all days (all PMETA < 0.05). Finally, translational effect sizes indicated less than “small” superiority on Day 4 (SMD 0.15, OR 0.78, NNT 23), and “small-medium-sized” superiority on Days 7 and 14 (Day 7: SMD 0.31, OR 0.59, NNT 12; Day 14: SMD 0.37, OR 0.53, NNT 10) for pain measured by VAS, as compared to diclofenac gel.

**Conclusion:**

The IPD meta-analysis of the TAASS and TRAUMED trials confirms the efficacy of Tr14 gel. In-depth analysis reveals it is a statistically favourable treatment with respect to pain, and function, compared to the gold-standard diclofenac gel. Taking this together, Tr14 gel represents a statistically favourable treatment option for Grade I–II ankle sprain within the context of the analysed endpoints.

**Supplementary Information:**

The online version contains supplementary material available at 10.1186/s12891-026-09802-0.

## Introduction

Lateral ankle sprains (LAS) are the most common injury in physically active individuals accounting for 3–5% of A&E admissions in the United Kingdom [1–3]. Prior history of LAS heightens its prevalence with 34% of cases experiencing re-injury within one year [4]. In the United States, 70% of individuals who sustain a LAS will suffer a degree of residual physical disability irrespective of their sporting level, or physical activity status [5, 6].

Contributory factors that increase the risk of further ankle injury include chronic ankle instability (CAI) and the development of post-traumatic osteoarthritis (PTOA) [7, 8]. Unresolved inflammation is understood to play a role in the chronicity of musculoskeletal injuries and may also have implications for ankle re-injury [9]. A study in patients undergoing LAS rehabilitation suggests that CAI will occur in 40% of individuals by 12-months [10]. While early prediction of CAI is unreliable in LAS, the authors identified several relevant factors present at 6-months post injury. These included a lower self-reported functional everyday living score (FAAM) alongside poorer dynamic postural control, indicative of a sensorimotor deficit (assessed via STAR [10]). Altered sensorimotor function is key contributor to patient-perceived instability following LAS and is understood to be triggered by pain and inflammation [11–13].

Historically, Non-Steroidal Anti-Inflammatory Drugs (NSAIDs) are first-line treatments for musculoskeletal injury-related inflammation, though their suitability is not without limitation. NSAIDs exert pain relief by dampening the initial inflammatory response via inhibition of the COX-1/2 pathway and thus prostaglandin production [14]. Their prolonged use compromises the integrity of gastrointestinal (GI) mucosa and may increase cardiovascular events restricting tolerability in those over 65 years of age, and in patients with related health conditions [1, 15]. Additionally, NSAIDs initiate molecular interactions that have implications for the resolution phase of inflammation [14]. These molecular disruptions may explain the clinically observed impairment in tendon healing following NSAID administration, as documented in both human and murine models of musculoskeletal injury [16–18]. These findings have prompted many clinicians to recommend the use of alternative analgesics with fewer side effects, like acetaminophen [12, 19]. Moreover, a recently published commentary addresses the mounting evidence that NSAIDs are disruptive to post-surgical healing. Authors call for further research to improve surgical practice, avoiding negative long-term consequences associated with routine NSAID use such as chronic post-surgical pain [20].

Pro-resolution pharmacology has practical applications in the management of musculoskeletal injury and is regulated by pro-resolution mediators (SPMs) associated with the fine orchestration of acute inflammation; initiation, transition, resolution, repair and return to homeostasis [21–23]. By interfering with their lipid mediator ‘class-switching’ abilities, NSAIDs are thought to inhibit the pro-resolving actions of SPMs [24]. Clinical studies suggest that NSAIDs can negatively impact SPM regulation following exercise-induced muscle damage, and their dysregulation of SPMs are associated with chronicity in lower back pain [25–27]. Efforts to identify therapeutics that enhance pro-resolution healing continue to grow [22] and Tr14 (Traumeel, Heel GmbH, Baden-Baden, Germany), a multicomponent formula of 14 primarily plant-based extracts, has demonstrated pro-resolving properties in several preclinical studies. In a murine model of peritonitis, Tr14 promoted SPM biosynthesis and efferocytotic macrophage activity to accelerate inflammation resolution [28]. In a murine skin wound-healing model, Tr14 demonstrated an upregulation of apoptosis, efferocytosis, and the M2 macrophage behaviour phenotypes in wounded skin samples. In contrast, diclofenac downregulated these cellular processes, highlighting the neutrophil-macrophage signalling axis as central to Tr14’s pro-resolving mechanism of action [29]. In novel approaches to injury, such as soft tissue engineering (STE), treatments that support pro-resolution signalling to enhance healing are therapeutically promising [30]. A role for immunoresolvents in tissue repair has been exemplified in mice, whereby administration of Resolvin D1 (RvD1) therapy reduced age-associated muscle fibrosis, and improved muscle function [31].

Growing clinical evidence highlights Tr14 as a viable alternative to topical NSAIDs in the treatment of LAS with data to indicate a shorter healing time, faster pain resolution, and few side effects [32, 33]. However, the most recent TRAUMED trial highlights Tr14’s potential superiority, as compared to the gold standard NSAID diclofenac [33]. This observation suggests Tr14 as a potential *superior* alternative to diclofenac treatment following acute LAS injury, in respect to pain and function. Here, we seek to examine this exploratory finding in closer detail. To achieve a more comprehensive understanding and generate more robust conclusions, one additional RCT with individual patient data (IPD) was available for confirmation and formal meta-analysis. Both trials, TAASS [32] and TRAUMED [33], investigate the efficacy of topical Tr14, versus topical 1% diclofenac, in the treatment of moderate to severe pain associated with acute LAS. Importantly, both studies adhere well to FAIR checklist guidelines demonstrating clinical and scientific integrity [34].

Meta-analytic techniques are recognised as a useful tool to summarise the overall efficacy results of a drug application (ICH Topic E 9 Statistical Principles for Clinical Trials, CPMP/EWP/2330/99) [35]. Given the lack of individual patient data analyses evaluating the overall efficacy of Tr14, this meta-analysis aims to combine two comparable datasets to better understand if Tr14 is significantly superior to the current gold standard topical NSAID treatment, diclofenac, in achieving pain resolution and functional improvement in patients after a Grade I/II lateral ankle sprain.

## Materials and methods

### Individual Patient Data (IPD) meta-analysis

#### General approach within the framework of this meta-analysis

This IPD meta-analysis was undertaken as a focused verification of a question arising from the TRAUMED study, namely whether applying the same eligibility criteria and analytic approach would yield consistent effects in an independent RCT dataset (TAASS), followed by synthesis of trial-specific estimates. It is therefore not positioned as a *de novo*, prospectively protocol-driven systematic review intended to identify all topical-therapy trials in the field. Reporting was informed by PRISMA-IPD. As an initial step, both individual participant datasets were independently validated by reproducing the original analyses and comparing them against the corresponding Clinical Study Reports using each study’s original methodology. Variables were then harmonised by applying consistent derivation rules and the prespecified methodological framework of the TRAUMED study to ensure comparable endpoint and covariate definitions across trials. For synthesis, we used a two-stage IPD approach: treatment effects were first estimated within each trial using the prespecified baseline-adjusted model and then pooled across trials using fixed-effect methods. Given that only two directly comparable trials were available, we emphasise trial-stratified estimates alongside pooled results.

A formal Individual Patient Data (IPD) meta-analysis was performed to compare efficacy of Tr14 gel with diclofenac gel [36]. The pre-specifications used in the most recent TRAUMED trial [33] were employed, including pain eligibility criteria and primary endpoints. The blinded methodological a priori operationalisations of the TRAUMED trial were equally applied to both trials to enhance harmonisation of endpoints across datasets. For TAASS, this constitutes a retrospective AUC reanalysis, which we acknowledge as a limitation and therefore trial-stratified results are emphasised alongside pooled estimates.

The primary endpoint for confirmatory analysis was the Area Under the Curve (AUC) for pain assessed using a visual analogue scale (VAS) from baseline to Day 4, and to Day 7 (a priori ordered hypotheses). Secondary endpoints, as far as available in both trials, were the *Foot and Ankle Ability Measure* (FAAM), *Activities of Daily Living* (ADL) subscale, time to 50% pain reduction, and the need for rescue medication.

Both trials used random sequence generation, allocation concealment, blinding of participants and staff, and blinding of outcome assessment. No incomplete outcome data of selective reporting was detected. Both studies underwent a rigorous centralised risk-based monitoring according to the FDA [37] and EMA Guideline [38] to ensure maximum data integrity and completeness. Dropout rate at the two primary endpoints Day 4 and Day 7 (see above) were below 1%, and risk of bias, as assessed by means of the Cochrane Risk of Bias tool (selection, performance, detection, attrition, reporting bias, and other bias) was low (Supplementary Table 1) [39, 40].

#### IPD analysis of single clinical trials - statistical methods

The IPD analysis followed a two-stage approach. Within each trial, the pre-planned primary procedure was an Analysis of Covariance (ANCOVA) on the primary endpoints with adjustment for baseline VAS pain scores. The pre-planned nonparametric sensitivity procedure was performed by means of the Wilcoxon-Mann-Whitney test (WMW) [41–44]. The effect size measure associated with the WMW test is the Mann-Whitney (MW) measure of superiority, a highly robust effect size measure with minimized assumptions representing the gold standard for full scale ordinal analysis [45–49] The technical expression for the MW is [P(X < Y) + 0.5 P(X = Y)]. The traditional benchmarks for the MW effect size measure are: [50, 51] 0.29 = large inferiority; 0.36 = medium inferiority; 0.44 = small inferiority; 0.50 = equality; 0.56 = small superiority; 0.64 = medium superiority; 0.71 = large superiority. Time-to-event analysis was performed by means of the Peto-Logrank test with Kaplan-Meier analysis. For event data, 2 × 2 tables with Fisher exact test were applied.

### Outcome measures

The IPD analyses and the subsequent meta-analyses were conducted on the following pre-specified primary outcome measures:

#### Primary Efficacy Variables (Confirmatory TRAUMED endpoints)

- AUC for VAS pain scores from baseline to Day 4.
- AUC for VAS pain scores from baseline to Day 7.

Secondary IPD endpoints were as follows:

#### Secondary Efficacy Variables (as far as available in both trials)

- AUC for VAS pain scores from baseline to Day 14.
- FAAM-ADL subscale on Day 4, 7, 14.
- Time to 50% persistent improvement of pain measured by VAS at each patient visit.

Regarding pain and function, percent changes from baseline were calculated in addition to ANCOVA analyses as robust sensitivity analyses, adjusting for proportional decreases. A further sensitivity analysis was conducted on interfering rescue medication (number of patients consuming paracetamol within the critical time window of 24 h prior to the primary pain measurements).

FAAM-ADL is commonly interpreted such that higher scores indicate better function, with the original scale ranging from 0 to 84 (21 items scored 0–4). For comparability, FAAM-ADL scores are often standardised to a 0–100 scale, with higher values indicating better function.

However, in the TRAUMED trial, FAAM-ADL changes from baseline were analysed both as absolute values and as percentage changes. To ensure consistent interpretability across endpoints and to avoid undefined percentage changes in the presence of baseline values of zero, FAAM-ADL scores were re-expressed by subtracting from the maximum possible score, such that lower values indicate better function.

This linear transformation preserves between-group comparisons while aligning the direction of benefit across outcomes. In this analysis, FAAM-ADL directionality is harmonised across TAASS and TRAUMED such that lower values consistently indicate better function (standardised scale: 0 = best, 100 = worst).

Associated effect measures:

- VAS pain score: mean difference (AUC ANCOVA), Mann-Whitney estimator (Percent-changes-from-baseline).
- FAAM-ADL: mean difference (ANCOVA), Mann-Whitney estimator (Percent-changes-from-baseline).
- Time to 50% persistent improvement in pain: mean difference (ANCOVA).
- Interfering rescue medication: Peto Odds Ratio.

### Treatment of missing values

The pre-specified handling of missing values was the Last-Value-Carried-Forward (LOCF) technique. This was retained to preserve the pre-specified missing-data approach of both parent trials and to ensure harmonization across the analyses of the two IPD datasets.

### Points in time

All points in time, as far as available in both trials, were used for the IPD analyses (Day 4, 7, and 14). The pre-defined primary points in time for outcome assessment were Day 4 and Day 7.

### Patient populations

Throughout this study, the pre-specified first line analysis of Tr14 compared to active control was based on the per protocol (PP) population. This approach reflects the non-inferiority heritage of the active-control objective; however, because the present analysis also discusses superiority (mean-difference analyses), we interpret these statements only where conclusions are supported across PP and the intention to treat (ITT)-concordant Full Analysis Set (FAS) sensitivity analysis. This definition was in line with the recommendations of the ICH E9 Guidance [52]for non-inferiority approaches. The PP population included all patients who were eligible for the full analysis set (FAS) evaluation and who additionally did not show significant protocol deviations (major protocol violations not related to death, adverse events, treatment failure, or good recovery). In both trials, all PP exclusions were pre-specified under blinded conditions in the final statistical analysis plan with individual listing of excluded patients. A sensitivity analysis of the primary endpoint pain AUC Day 4 and Day 7 was performed based on the FAS to evaluate the robustness of the PP results. The FAS sensitivity analysis reflects the ITT principle, with only four out of 650 randomized patients of the meta-analysis excluded from FAS analysis (all four patients lacked any kind of follow-up efficacy data).

### Methods of synthesis

The pre-planned method of synthesis for the outcome ensembles of the two single trials were the classic pooling procedures based on the fixed effect model (Hedges-Olkin) [53]. Generation of a funnel plot (Egger) [54] to assess risk of publication bias was omitted since the number of included studies was only 2, i.e. below the minimum number of 10 studies recommended for funnel plot analysis (Cochrane Handbook for Systematic Reviews of Interventions) [55]. Tests for quantitative heterogeneity of the two studies were performed using standard chi-square statistic and I^2^ statistic [56].

For the synthesis of the robust nonparametric analysis of percent-changes-from baseline, the Wei-Lachin test of stochastic ordering (one-dimensional test) [57] was applied in addition, a maximin-efficient robust test (MERT) [58, 59] which provides an overall MW estimate and test of treatment effect from an ensemble of independent studies. In combination with stochastic ordering or a one-dimensional alternative, it is a powerful and robust meta-analytic procedure for combining the MW effect size across studies. The one-dimensional alternative of stochastic superiority is to be interpreted as follows: at least one trial has an underlying true beneficial effect, and none have an adverse effect (no qualitative interaction). In contrast to other pooling procedures, the Wei-Lachin procedure is also appropriate where only limited studies are included, such as in the present situation involving two studies of identical design. Originally developed for robust pooling of subgroup results, the Wei-Lachin approach requires minimal assumptions and is also robust in the presence of heterogeneity [57]. Qualitative interaction of the studies was tested using the Gail-Simon test [60]. See Supplementary Methods S1, for a “Plain Language Summary of Methods of Synthesis”.

### Characteristics of the studies included in the meta-analyses

#### TAASS^1^

This prospective, randomised, controlled, multi-center, two-stage (Bauer-Köhne) [61] confirmatory clinical trial compared the efficacy of Tr14 gel in terms of pain and function with a topical NSAID (diclofenac) in athletes with acute unilateral ankle sprain. An additional group of patients received Tr14 ointment [single-blind], however, this treatment is not part of the present meta-analysis. Included were males/females aged from 18 to 40 years who had suffered an acute unilateral sprain of the lateral ankle joint within 24 h of the first dose of study medication. To be eligible, all participants had to be experiencing moderate (30–60 mm) to severe (> 60 mm) pain on weight-bearing (using a self-assessed VAS pain score) and be unable to perform their normal training/sports activities. In addition, participants provided informed consent and were available for the duration of the study.

The total individual treatment period was 2 weeks (first patient enrolled: 24.08.2009, last patient completed: 12.09.2012). The study was double-blind for Tr14 gel and diclofenac gel. Treatment involved application and gently rubbing of 2 g gel three times daily, to sufficiently cover the affected area. Randomisation was performed by means of sealed allocation. Investigators, assessors, and site personnel were blinded as to the allocated study treatment. The primary efficacy criterion was the patient’s assessment of their ankle pain on weight bearing using a VAS. The primary time point of interest in the TAASS trial was Day 7. Additional efficacy criteria available for meta-analysis, were the FAAM-ADL subscale, time to 50% pain reduction, and use of rescue medication.

For maximum consistency, the TAASS IPD analysis was performed as pre-specified in the final SAP of the more recent TRAUMED trial. This meant applying (1) the stricter TRAUMED pain inclusion criterion of at least 50 mm on the VAS (see section *Material and Methods*,* Characteristics of the studies included in the meta-analyses*,* TRAUMED*), as well as (2) performing the primary TRAUMED AUC analysis with baseline adjustment.

By using the above methodology, the IPD analyses of the two trials followed identical pre-specified rules, thus preventing selection or methodological bias. Demographic criteria and other baseline characteristics of the TAASS IPD population are provided in Table 1.

**Table 1:** TAASS-IPD population - demographic criteria and other baseline characteristics; Tr14 vs. Diclofenac

**Primary IPD Population**							
Baseline characteristic		Tr14		Diclofenac			Effect size
		*N* = 80		*N* = 88			Mann-Whitney^1^)
Age (Years)	Mean (SD, Range)	26.3(6.17, 18–40)		26.4 (6.22, 18–40)			0.4969
Gender	Male	57	(71.3%)	65	(73.9%)		0.5131
	Female	23	(28.8%)	23	(26.1%)		
BMI (kg/m^2^)	Mean (SD, Range)	23.2 (2.67, 18.6–30.9)		23.2 (2.42, 17.3–30.7)			0.4786
Injury							
Grading	Grade 1	43	(53.8%)	40		(45.5%)	0.4559
	Grade 2	37	(46.3%)	47		(53.4%)	
	Grade 3	0	(0.0%)	1		(1.1%)	
Efficacy							
VASWB	Mean (SD, Range)	66.6 (12.35, 51–95)		63.3 (10.1, 51–95)			0.4317
FAAM	Mean (SD, Range)	46.9 (20.05, 7–95)		51.5 (17.90, 17–95)			0.5802

*Abbreviations*: *Tr14* Traumeel^®^, *IPD* Individual Patient Data (Per-Protocol analysis set), *N*  Number of patients, *Mean* arithmetic mean, *SD* Standard Deviation, *BMI*  Body Mass Index, *VASWB* VAS pain score on Weight Bearing, *FAAM* Foot and Ankle Ability Measure - Activities of Daily Living (21-item self-report questionnaire subscale)

^1)^ The Mann-Whitney estimator is the corresponding standardised effect size measure of the Wilcoxon-Mann-Whitney test, benchmarks: 0.5 equality, 0.44/0.56 small, 0.36/0.64 medium-sized, 0.29/0.71 large group difference

### TRAUMED^2^

The TRAUMED trial was a prospective, randomised, controlled, multi-center, active- and placebo-controlled, confirmatory clinical trial to demonstrate efficacy and safety of Tr14 Gel in patients having suffered an acute lateral ankle injury. Included were male or female outpatients from 18 years of age (*range:18–78 years*), with a unilateral Grade 1 or Grade 2 sprain occurring within 24 h of first treatment. The primary outcome measure was pain > 50 mm (assessment after 5 min rest) on passive movement determined by the investigator using a VAS.

The total individual treatment period was 7 days followed by another 7 days treatment-free follow-up phase. Final assessment occurred 2 weeks after enrolment (first patient enrolled: 26.02.2018, last patient completed: 18.11.2020). Randomisation was performed by means of sealed allocation. Investigators, assessors, and site personnel were blinded to the allocated study treatment. The a priori ordered primary efficacy criteria (fixed sequence) [62] were AUC* for pain on passive movement on VAS from baseline to Day 4, and to Day 7. Additional efficacy criteria, available for meta-analysis in both trials, were the FAAM-ADL subscale, time to 50% pain reduction, and consumption of rescue medication. The pre-specified confirmatory dataset for comparison with the active control (diclofenac) was the PP population. Demographic criteria and other baseline characteristics of the IPD population (identical with the primary confirmatory dataset for comparison to active control) are provided in Table 2.

**Table 2:** TRAUMED trial population: demographic criteria and other baseline characteristics; Tr14 vs. diclofenac

**Primary IPD Population**						
Baseline Characteristic		Tr14		Diclofenac		Effect Size
		*N* = 314		*N* = 146		Mann-Whitney^1^)
Age (Years)	Mean (SD, Range)	35.1(14.11, 18–78)		34.1 (13.19, 18–75)		0.5147
Gender	Male	160	(51.0%)	86	(58.9%)	0.5397
	Female	154	(49.0%)	60	(41.1%)	
Ethnic	Caucasian	303	(96.5%)	144	(98.6%)	0.5106
Origin	Black	3	(1.0%)	0	(0.0%)	
	Asian	1	(0.3%)	0	(0.0%)	
	Other	7	(2.2%)	2	(1.4%)	
BMI (kg/m^2^)	Mean (SD, Range)	26.2 (4.66,18.9–45.9)		24.8 (4.11, 17.8–39.5)		0.5848
Injury						
Grading	Grade 1	215	(68.5%)	107	(73.3%)	0.5241
	Grade 2	99	(31.5%)	39	(26.7%)	
	Grade 3	0	(0.0%)	0	(0.0%)	
Efficacy						
VASPM	Mean (SD, Range)	74.9 (10.99, 51–100)		75.2 (11.18, 53–100)		0.5102
FAAM	Mean (SD, Range)	51.3 (17.37, 0–95)		50.4 (18.76, 0–98)		0.4838

Abbreviations: *Tr14* Traumeel^®^, *IPD* Individual Patient Data (Per-Protocol analysis set), *N *Number of patients, *Mean *arithmetic mean, *SD* Standard Deviation, *BMI* Body Mass Index, *VASPM* VAS pain score on Passive Movement, *FAAM* Foot and Ankle Ability Measure - Activities of Daily Living (21-item self-report questionnaire subscale)

^1)^ The Mann-Whitney estimator is the corresponding standardised effect size measure of the Wilcoxon-Mann-Whitney test, benchmarks: 0.5 equality, 0.44/0.56 small, 0.36/0.64 medium-sized, 0.29/0.71 large group difference

## Results

A total of 628 patients were included in the primary IPD meta-analysis of the two trials. The rate of premature discontinuations before any of the two primary endpoints Day 4 and Day 7 (see section *Material and Methods*,* Points in Time*) were below 1% (TAASS: 4/168; TRAUMED: 1/460). The mean age of the patients was 26.3 years (TAASS), and 34.8 years (TRAUMED). The proportion of males was 72% and 53%, and the total pain VAS score pre-treatment was 65 mm and 75.0 mm, for TAASS and TRAUMED studies, respectively. Further characteristics of the included trials and patients are described in Tables 1 and 2.

### PAIN VAS

**Fig. 1:**
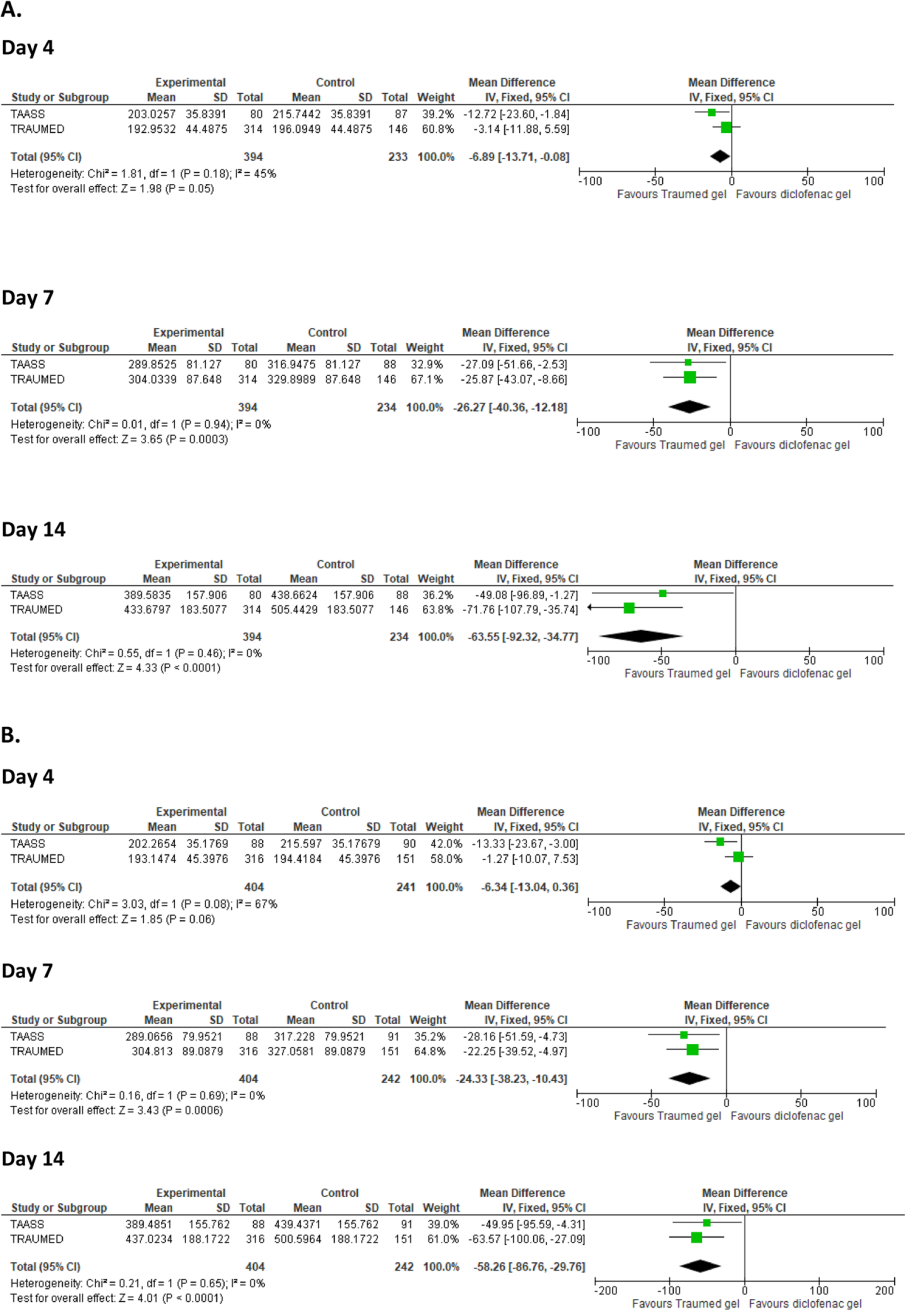
Forest Plot of Comparative VAS Pain Scores (AUC) & Sensitivity Analysis on FAS-LOCF. Following exposure to Experimental (Tr14), or Control (diclofenac gel), VAS pain scores were measured on Days 4, 7, and 14, and were analysed via AUC ANCOVA applied to IPD with PP-LOCF (**A**) and FAS-LOCF to assess for sensitivity (**B**). Effect sizes with their associated confidence interval are expressed as ‘Mean Difference’ for TAASS (small green square), TRAUMED (large green square), and Total (black rhombus). Abbreviations: Traumed gel (Tr14 gel), VAS (Visual Analogue Scale), ANCOVA (Analysis of Covariance), IPD (Individual Patient Data), PP (Per-Protocol), LOCF (Last Value Carried Forward), IV (Inverse Variance), Fixed (Fixed Effect Model), CI (Confidence Interval), SD (Standard Deviation)

Tr14 gel significantly improved pain VAS scores in the primary AUC ANCOVA analysis as compared to diclofenac gel (Day 4 mean difference MD_AUC−META_ = -6.9 [95%CI -13.7 to -0.1], P_AUC−META_ = 0.05; Day 7 MD_AUC−META_ = -26.3 [95%CI -40.4 to -12.2], P_AUC−META_ = 0.0003; Day 14 MD_AUC−META_ = -63.6 [95%CI -92.3 to -34.8]), P_AUC−META_ < 0.0001; Fig. 1.A). There was no indication for relevant heterogeneity of the trials regarding the primary endpoints (all I^2^ < 50% with associated *P* > 0.1). In the FAS sensitivity analysis (LOCF), the pooled estimates were consistent with the PP analysis (Day 4 mean difference MD_AUC−META_ = − 6.34; Day 7 MD_AUC−META_ = − 24.33; Day 14 MD_AUC−META_ = − 58.26; Fig. 1.B, supporting robustness of the PP-LOCF primary analysis. Adjusted means of absolute pain VAS scores for Tr14/diclofenac were 36.6/42.9 (Day 4), 21.5/25.0 (Day 7), and 7.0/9.8 (Day 14) in the TAASS trial, and 44.3/49.5 (Day 4), 26.3/33.7 (Day 7), and 11.0/16.4 (Day 14) in the TRAUMED trial (lower values indicating less pain). Translational effect sizes indicated less than “small” superiority, as compared to diclofenac gel, on Day 4 (SMD 0.15, OR 0.78, NNT 23), and “small” to “medium-sized” superiority on Day 7 and Day 14 (Day 7: SMD 0.31, OR 0.59, NNT 12; Day 14: SMD 0.37, OR 0.53, NNT 10) (Supplementary Table 2).

**Fig. 2:**
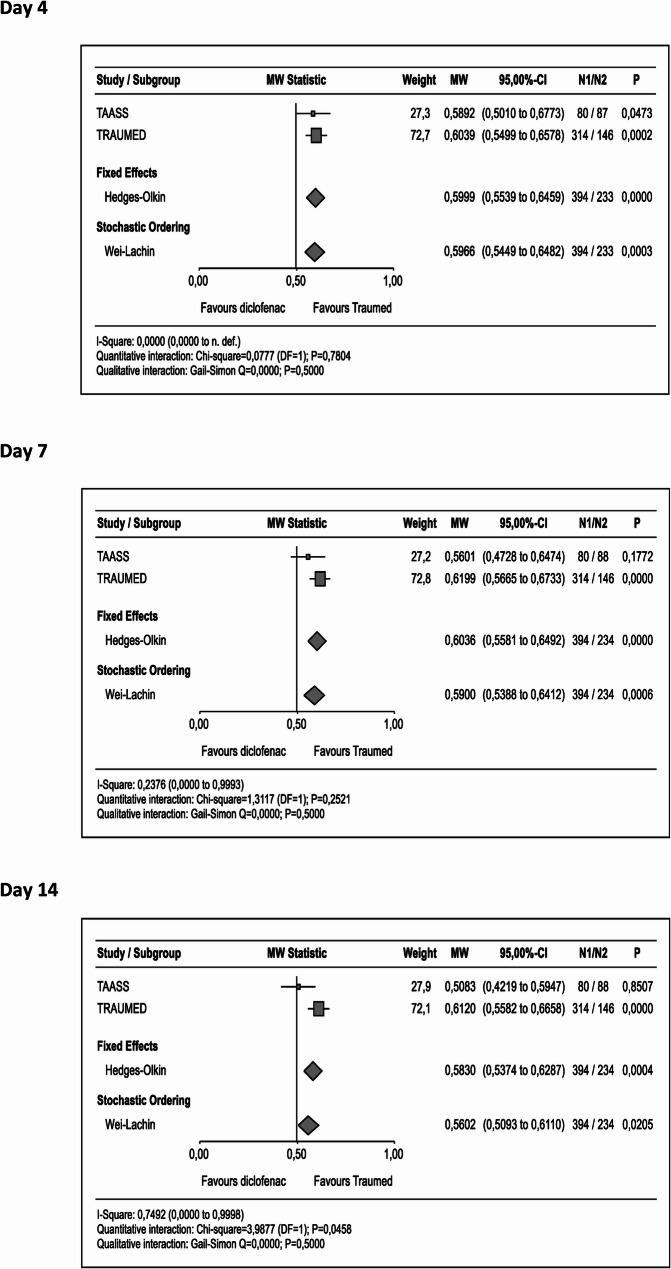
Forest plot of comparative VAS pain scores (percent changes from baseline). Following exposure to Experimental (Tr14 gel), or Control (diclofenac gel), percent changes from baseline calculated from VAS pain scores measured on Days 4, 7, and 14, were analysed via a Wilcoxon-Mann-Whitney test (applied to IPD with PP-LOCF). Effects sizes with their associated confidence interval are expressed by means of the ‘Mann-Whitney Statistic’ for TAASS (small square), TRAUMED (large square), Fixed Effect (top rhombus), and Stochastic Ordering (lower rhombus). Abbreviations: Traumed (Tr14 gel), MW (Mann-Whitney), see Fig. 1 for additional abbreviation expansion

The analysis of the percent-changes-from-baseline confirmed the superiority of Tr14 gel with respect to pain reduction on all visit days (Day 4 MW_META_ = 0.60 [95%CI 0.55 to 0.65], P_META_ <0.0001; Day 7 MW_META_ = 0.60 [95%CI 0.56 to 0.65], P_META_ <0.0001; Day 14 MW_META_ = 0.58 [95%CI 0.54 to 0.63], P_META_= 0.0004; Fig. 2). Median percent decreases were − 41.9%/-35.6% (Day 4), -71.7%/-64.3% (Day 7), and − 93.3%/-93.3% (Day 14) in the TAASS trial, and − 39.6%/-28.2% (Day 4), -68.9%/-55.7% (Day 7), and − 93.3%/-85.0% (Day 14) in the TRAUMED trial. All in all, the meta-analysis on pain VAS scores provides good evidence for positive effects of Tr14 gel as compared to diclofenac gel.

### Foot and Ankle Ability Measure - Activity of Daily Living (FAAM-ADL)

**Fig. 3:**
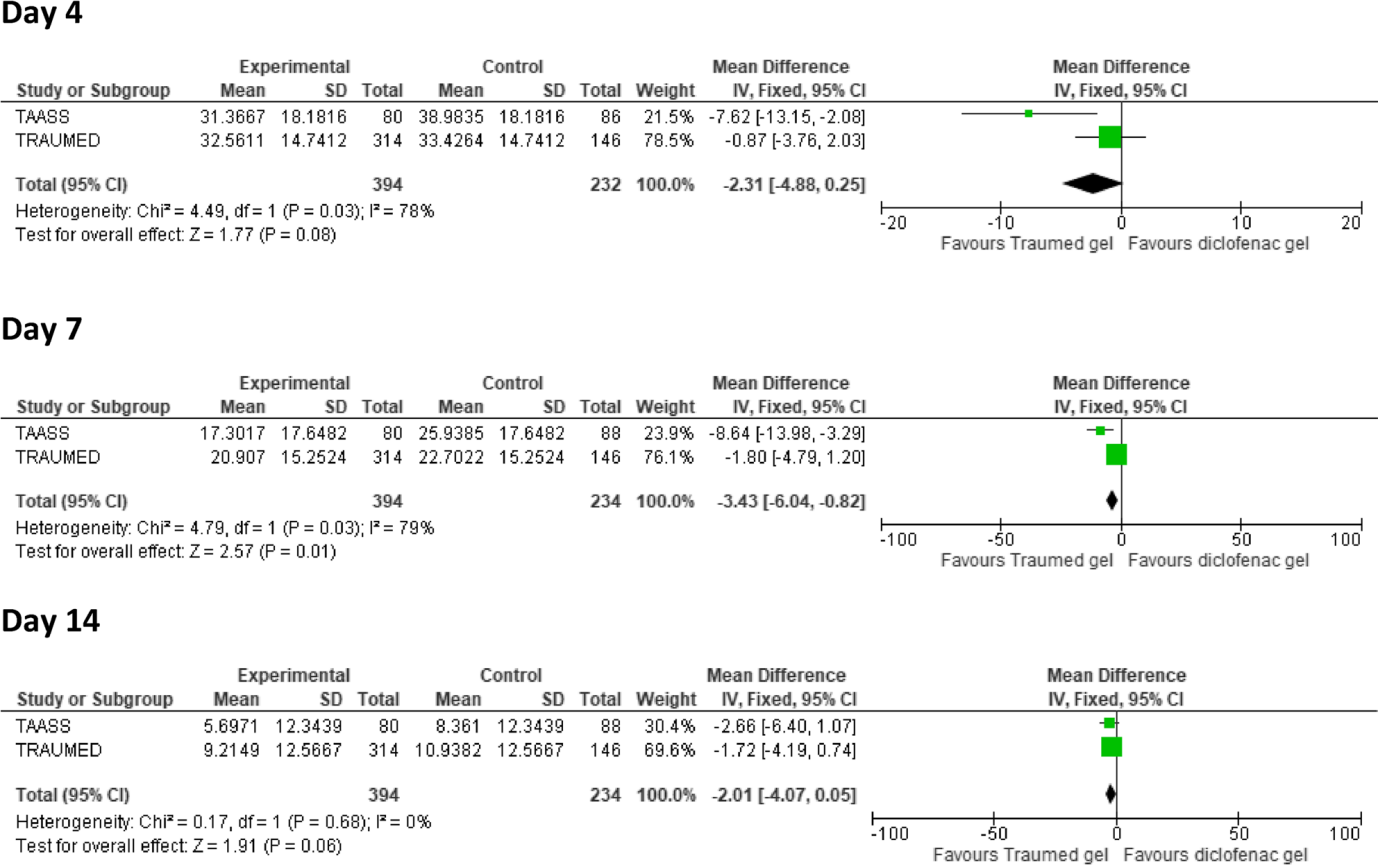
Forest Plot of Comparative FAAM-ADL Scores (ANCOVA). Following exposure to Experimental (Tr14 gel), or Control (diclofenac gel), absolute FAAM scores measured on Days 4, 7, and 14, and were analysed via ANCOVA (applied to IPD with PP-LOCF). Effect sizes with their associated confidence interval are expressed as ‘Mean Difference’ for TAASS (small green square), TRAUMED (large green square), and Total (black rhombus). Note: FAAM-ADL direction was harmonised so that lower values indicate better function (less impairment). Abbreviations: Traumed gel (Tr14 gel), FAAM-ADL (Foot and Ankle Ability Measure - Activities of Daily Living (21-item self-report questionnaire subscale)), see Fig. 1 for additional abbreviation expansion

Based on the ANCOVA analyses of the absolute FAAM scores, the meta-analysis showed improved joint function following application of Tr14 gel, as compared to diclofenac gel, with statistically significant superiority on Day 7 (Day 4 P_META_ = 0.08, Day 7 P_META_ = 0.01, Day 14 P_META_ = 0.06; Fig. 3). Adjusted means of absolute FAAM scores for Tr14/diclofenac were 31.4/39.0 (Day 4), 17.3/25.9 (Day 7), and 5.7/8.4 (Day 14) in the TAASS trial, and 32.6/33.4 (Day 4), 20.9/22.7 (Day 7), and 9.2/10.9 (Day 14) in the TRAUMED trial (lower values indicate better function; FAAM direction was harmonised across trials as described in Methods).

**Fig. 4:**
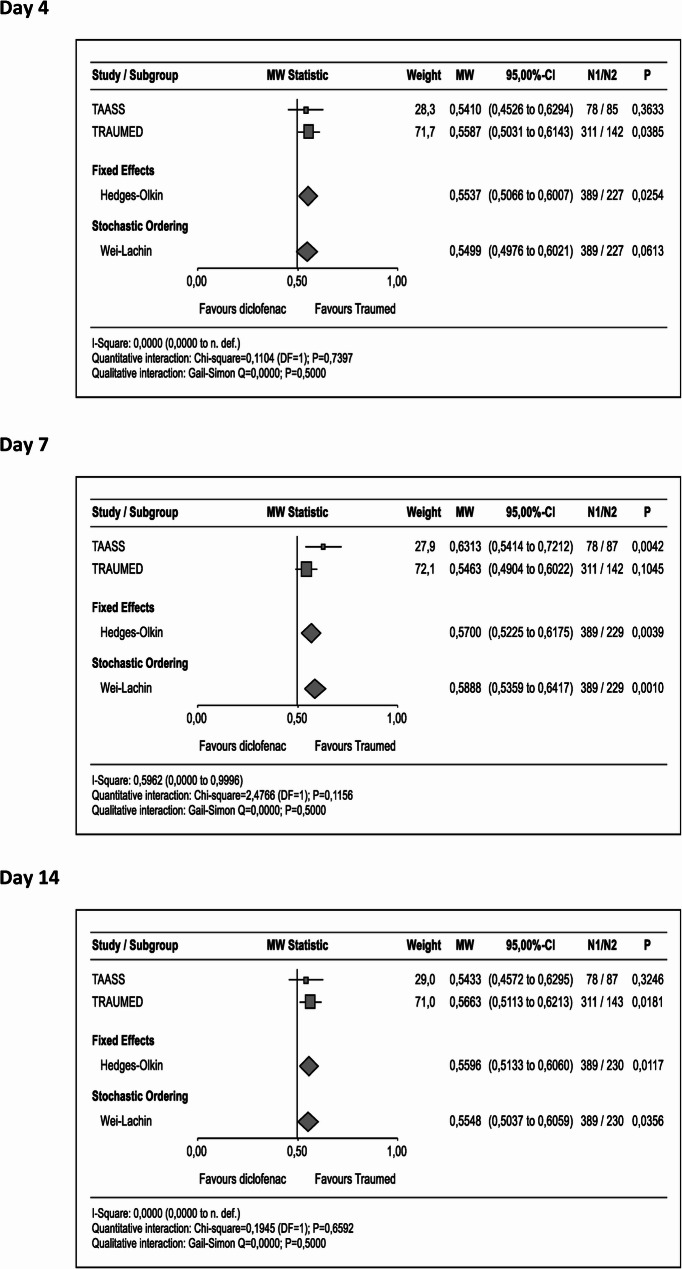
Forest Plot of Comparative FAAM-ADL Scores (Percent Changes from Baseline). Following exposure to Experimental (Tr14 gel), or Control (diclofenac gel), percent changes from baseline calculated from FAAM-ADL scores measured on Days 4, 7, and 14, were analysed via a Wilcoxon-Mann-Whitney test (applied to IPD with PP-LOCF). Effects sizes with their associated confidence interval are expressed by means of the ‘Mann-Whitney Statistic’ for TAASS (small square), TRAUMED (large square), Fixed Effect (top rhombus), and Stochastic Ordering (lower rhombus). Note: FAAM-ADL direction was harmonised so that lower values indicate better function (less impairment). Abbreviations: Traumed (Tr14 gel), FAAM-ADL (Foot and Ankle Ability Measure - Activities of Daily Living (21-item self-report questionnaire subscale)), MW (Mann-Whitney), see Fig. 1 for additional abbreviation expansion

The robust nonparametric analysis of the percent-changes-from-baseline measured by the FAAM-ADL for Tr14 shows superiority of Tr14 vs. diclofenac on all visits (Day 4 MW_META_ = 0.55 [95%CI 0.51 to 0.60], P_META_=0.0254, Day 7 MW_META_ = 0.57 [95%CI 0.52 to 0.62], P_META_=0.0039; Day 14 MW_META_ = 0.56 [95%CI 0.51 to 0.61], P_META_=0.0117, Fig. 4). Thus, regarding joint function, there is good indication for positive effects of Tr14 gel as compared to diclofenac gel.

### Time to 50% improvement in VAS pain scores

**Fig. 5:**
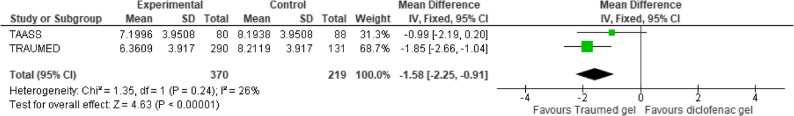
Forest Plot of Comparative Time to 50% Pain Decrease. Following exposure to Experimental (Tr14 gel), or Control (diclofenac gel), available records measuring Time (days) until 50% decrease in pain were analysed via ANCOVA (applied to available IPD, with PP). Effect sizes with their associated confidence interval are expressed as ‘Mean Difference’ for TAASS (small square), TRAUMED (large square), and Total (black rhombus). Abbreviations: Traumed gel (Tr14 gel), see Fig. 1 for additional abbreviation expansion

Regarding the time to 50% improvement, the combined baseline-adjusted mean difference (MD_META_) showed a difference of 1.6 days with respect to the time to 50% improvement, favoring Tr14 gel (MD_META_ = -1.58 [95%CI -2.25 to -0.91], P_META_ < 0.00001, Fig. 5). While 99% of the patients reached at least a 50% pain decrease within the 14-day period, indicating a negligible number of *censored* values (2 out of 370 patients in the Tr14 group (0.5%), and 5 out of 219 patients in the diclofenac group did not achieve such improvement (2.3%)).

### Rescue medication

In both trials, paracetamol (acetaminophen, 500 mg/tablet) was provided as rescue medication with daily entry of tablet intake in the patient’s diary. Requirement for rescue medication was low across groups at any point during the trials. In the TAASS trial, the adjusted mean of the total tablet consumption until Day 14 was 1.3 tablets in each of the two treatment groups. In the TRAUMED trial, the adjusted means of both groups were below 1 tablet for the total study duration.

To evaluate the potential influence of rescue medication intake upon pain VAS measurements during the critical pain-loaded primary visits, frequency counts were performed for the number of patients with rescue medication intake within the 24-hour window prior to the pain measurements on Day 4 and Day 7 (risk of interference). Regarding the comparison of Tr14 gel vs. diclofenac gel, the Individual Patient Data (IPD) analysis of the two trials provided no indication for differences regarding the interference rates (P_META_ > 0.4 for both visits; TAASS trial: the highest single interference rate was found with 3.4% at Day 4 in the diclofenac group; TRAUMED trial: the highest single interference rate was found with 1.0% at Day 4/7 in the Tr14 gel group). Thus, the influence of rescue medication on the primary pain measurements may be regarded as negligible, with a very low risk of bias.

## Discussion

The objective of this meta-analysis was to evaluate the efficacy of Tr14 gel compared to diclofenac gel in patients with moderate to severe pain (VAS ≥ 50 mm) suffering a Grade I-II LAS. The excellent baseline comparability between groups facilitated an examination of two active controlled Tr14 trials, TAASS [32] and TRAUMED [33]. The IPD analysis shows that the translational effect sizes of topical Tr14 gel for pain (measured by VAS) fall within established MCID thresholds, and that concordant PP and FAS sensitivity analyses further demonstrate statistically favourable outcomes for Tr14 gel over the current gold-standard topical treatment, diclofenac gel, with respect to both pain and function. These results support the therapeutic potential of Tr14 gel for the treatment soft tissue injuries.

LAS is the most prevalent musculoskeletal injury in physically active populations, accounting for 16–40% of all joint injuries [1]. An initial barrier to effective care is patient perception. Often, LAS is wrongly considered an inconsequential injury with 50% of those affected failing to seek formal clinical care: a factor contributing to its high recurrence rate [63, 64]. The substantial impact of diagnostic, inpatient, and hospital fees are well recognised but the challenge of indirect patient cost, such as additional childcare, are still emerging [65, 66]. Improved preventative and therapeutic strategies are warranted to manage LAS, limit its recurrence, and reduce the associated socio-economic burden [3]. Here, the meta-analysis showed a combined baseline-adjusted mean difference (MD_META_) of 1.6 days with respect to time to 50% improvement, favoring Tr14 gel. This may hold translational relevance in lessening the social costs associated with injury. A faster return to normal activity, by more than one day, could provide patients an opportunity to return to daily and sporting activity more quickly.

Non-inferiority of Tr14 gel, as compared to diclofenac gel, was confirmed in this meta-analysis at all endpoints on the pre-defined non-inferiority margin (MW: all LB-CI > 0.4, AUC: all LB-CI < 25). Importantly, the pre-defined MW margin of 0.4 was identical in both trials, in line with the German Regulatory Authority (BfArM) recommendations. Moreover, the non-parametric analysis, with its minimised assumptions and standardised effect sizes, independent of data type and scale, fully confirmed the raw-scale results.

In addition to non-inferiority, statistically favourable outcomes versus the active comparator were observed, contingent upon concordant findings in both PP and FAS analyses. As noted in the ICH E9 Biostatistics Guidance [52], “*efficacy is most convincingly established… by showing superiority to an active control treatment*”. In line with this statement, the meta-analysis establishes efficacy of Tr14 with respect to pain, at all measured time-points, as well as with respect to function (on day 7 for absolute scores, and on days 4, 7, and 14 for percent change), compared with 1% diclofenac gel. Moreover, Tr14 showed an increasing trend of superiority over diclofenac across time points: the initial less than “small” superiority observed on Day 4 became more evident by Day 7 and reached its highest level on Day 14. Thus, the statistical favourability of Tr14 gradually strengthened over time, peaking at Day 14. Furthermore, for pain measured via VAS, translational analysis well supports these effects on Days 7 and 10, where NNTs of 12 and 10, respectively, fall comfortably between the accepted benchmarks of ‘small’ (NNT: 18) to ‘medium’ (NNT: 7) clinically relevant effect sizes (Supplementary Table 2). In respect to function, clinically relevant effect sizes peak at Day 7 with translational effects sizes ranging between small-medium MCID benchmarks, NTT = 16 and NTT = 11, for ANCOVA and % Changes from Baseline FAAM-ADL, respectively.

Diclofenac is considered a gold standard NSAID in the treatment of joint sprain, and other conditions. Topical diclofenac has been demonstrated as efficacious in the short-term treatment of knee osteoarthritis in patients unable to tolerate systemic NSAIDs [67, 68]. However, there are reports of its poor efficacy in more diverse musculoskeletal pain, including chronic low back and neuropathic pain [69]. Avoiding first pass metabolism, topical preparations exert fewer systemic effects [70–72] but NSAIDS are understood to impede the natural healing process in soft tissue regardless of a systemic, or local, administration route [14, 16, 17].

Tr14 orchestrates its molecular response to pain and inflammation differently to NSAIDs. A recent analysis of tissue derived from a time-series wound-healing murine model demonstrated that, in contrast to diclofenac, pro-inflammatory gene expression was largely unchanged during inflammation initiation in Tr14- treated mice. However, notable differences in the resolution phases indicated that Tr14 upregulated several key processes associated with inflammation resolution, such as apoptosis, efferocytosis, and M2-like macrophage behavior [29]. This suggests Tr14 may have a distinct molecular advantage in inflammation initiation, essential to the resolution and restoration of tissue homeostasis. In contrast, NSAIDs suppress inflammation initiation and thus impede optimal healing [26].

The findings of this meta-analysis are well supported by earlier controlled trials demonstrating Tr14’s efficacy and tolerability in musculoskeletal injury. Historically, several authors report a reduction in pain and swelling, and improvements in the mobility of joints such as the ankle and knee [73, 74]. We found that Tr14 is effective in achieving faster pain resolution, shortening it by up to 1.6 days within a 14-day period. These results suggest there may be potential for Tr14 to alleviate some of the burden associated with LAS, including fewer health care interactions. Tr14 application may also expedite the return to sport (RTS) following injury, representing a critical advantage for those participating in time-sensitive, competitive events. Moreover, Tr14 has an excellent safety profile and is available over the counter, making it an easily accessible and safe treatment option. Future research into its therapeutic potential in other soft tissue inflammation disorders, particularly in tissues poorly responsive to NSAIDs, may be warranted.

It is not yet clear if Tr14’s early clinical efficacy may infer some protection against the development of chronic ankle instability, a common feature contributing to re-injury incidence in LAS [8, 75]. Future studies assessing the link between inflammation and sensorimotor deficit, a key contributor to CAI, will be useful [76]. Tr14’s potential to preserve sensorimotor function via its pro-resolution action also warrants further exploration. Thus far, studies measuring the effect of topical Tr14 (or diclofenac) on sensorimotor function do not exist.

### Strengths and limitations

A notable strength of this study is the statistical design employed to facilitate maximum methodological consistency across trials, ensuring the delivery of robust findings. Applying the final statistical analysis plan of the latest, and largest, trial (TRAUMED) [33] to both trials ensured the execution of IPD analysis. In turn, this avoided arbitrary selection while controlling for the inclusion range of pain. Moreover, to facilitate clinical interpretation and contextualise the findings with other studies, these results were additionally expressed as translational effect sizes, including standardized mean differences (SMD), Mann–Whitney measures (MW), odds ratios (OR), and numbers needed to treat (NNT) (see Supplementary Table 2) [49–51].

There are, however, several limitations. Firstly, this analysis is not a prospective meta-analysis (PMA). Two eligible head-to-head RCTs with accessible IPD were identified for this focused IPD meta-analysis (Supplementary Fig. 1), lessening the potential for study selection bias. Additionally, the rigorous implementation of the pre-specified TRAUMED rules prevented selection bias of outcome and analysis, respectively.

Secondly, patients with mild pain (VAS score < 50 mm), and those suffering a Grade III LAS were excluded. Therefore, we do not know whether Tr14 is beneficial across a wider spectrum of LAS severity. For example, symptoms may change, or recur, in the subsequent weeks outside of the research period. Thirdly, there was a difference in how pain was assessed between the two trials: in the earlier TAASS trial [32] pain was measured as *pain on weight bearing*, while in the TRAUMED study pain was measured as *pain on passive movement*. Both trials used a similar patient-determined 100 mm VAS to measure pain, but it is possible that the discomfort experienced when applying body weight to the affected ankle may be different than that caused by a physician stressing the joint. Should this be the case, discrepancies between the respective VAS scores are expected to be small and not of the magnitude encountered in the comparison between *pain on weight bearing* and *pain at rest*. This was exemplified in a recent ankle sprain study that reported a baseline pain on weight bearing of 8.4, compared to pain at rest of 6.5 (placebo means of 11-point numeric rating scale) [77]. This supports the interpretation that mechanically provoked pain measures differ from pain at rest but remain within a comparable range of clinical severity. Similar findings were encountered in the TRAUMED [33] trial, where scores for pain on passive movement were substantially higher than pain at rest, and these differences were of a similar magnitude in those reporting pain on weight bearing. These represent related but non-identical provocation conditions and are therefore interpreted carefully in pooled analyses; trial-stratified estimates are provided prominently in all analyses.

Fourth, the nature of the patient population may be regarded as a limitation. While in TAASS^1^ [32] only athletes were included, the TRAUMED [33]study was not limited to athletes, but rather included a more representative, slightly older population with differing degrees of LAS severity. To address this, a sensitivity analysis regarding the grade of ankle sprain was performed to obtain adjusted “like-to-like” comparisons, and to investigate potential subgroup effects. The stratified IPD analyses of the two strata of interest (“Grade 1” and “Grade > 1”) were performed on the primary criterion PAIN AUC ANCOVA, with subsequent meta-analytic pooling *within* each subgroup, formal pooling *across* subgroups, and provision of subgroup-specific statistics that included measures of heterogeneity. The combined subgroup-adjusted results well confirmed the findings of the combined main analyses (PAIN AUC ANCOVA [PP]: Day 4 P_AUC−META_ = 0.05; Day 7 P_AUC−META_ = 0.0003; Day 14 P_AUC−META_ < 0.0001; all tests for subgroup differences with *P* > 0.1 and I^2^ < 0.5; Supplementary Fig. 2).

A fifth limitation is patient heterogeneity towards the end of the 14-day assessment period. The robust, non-parametric analysis of the percent-changes-from-baseline, with its implicit baseline-adjustment, in case of proportional decrease, well confirmed the statistical favourability of Tr14 gel with respect to pain reduction at the primary visits Day 4 and Day 7. However, while heterogeneity was low at the two primary visits Day 4 (I^2^ = 0%) and Day 7 (I^2^ = 24%), there was substantial heterogeneity at the secondary endpoint Day 14 (I^2^ = 75%). This may well be explained by ‘floor effects’ on Day 14, due to lower baseline pain levels in the TAASS study preventing sufficient pain differentiation at this later point in time (TAASS mean pain VAS scores at baseline: 65 mm vs. TRAUMED mean scores at baseline 75 mm; percent-changes-from-baseline Day 14: P_perc−META_ = 0.0004).

A further limitation to consider is the use of the Last-Observation-Carried-Forward (LOCF) imputation method to account for missing data. We acknowledge that LOCF operates under restrictive assumptions and carries a theoretical risk of introducing bias or underestimating variance, particularly in pain studies characterised by improving trajectories. However, the dropout rate prior to the primary analytical endpoints (Days 4 and 7) in the pooled cohort was exceptionally low (less than 1%). Because this fraction of missing data was so small, the potential bias incurred by the LOCF strategy is mathematically negligible and renders advanced likelihood-based models, such as MMRM or multiple imputation, redundant.

A final potential limitation concerns the patient population chosen to analyse. The pre-specified first line analysis of the comparison of Tr14 to active control was throughout based on the PP population, due to the confirmatory primary non-inferiority objective of the TRAUMED [33] trial (see section *Material and Methods*,* Patient Populations*). This was in line with the recommendations of the ICH E9 Guidance [52]for non-inferiority approaches because the full analysis sets (FAS) in such trials are “generally not conservative”. A sensitivity analysis of the primary endpoint PAIN AUC, however, was performed based on the full analysis set (FAS) to evaluate the robustness of the PP results. The results were well comparable, attributable not least to the very low number of dropouts or protocol violators in both trials, suggesting patient population should not be an issue (Fig. 1.B).

## Conclusion

An Individual Patient Data (IPD) meta-analysis of two recent prospective, randomised, double-blind multicenter trials in patients with acute ankle sprain confirms the beneficial effects of Tr14. When compared to diclofenac, it resulted in statistically favourable pain relief and physical function, supported by concordant PP and FAS sensitivity findings. These results confirm that Tr14 is a suitable option for the treatment of moderate-severe lateral Grade I-II ankle sprain. Future investigations into the clinical relevance of Tr14 could benefit from placebo-controlled trials with extended observation periods to better evaluate its efficacy in specific populations with mild or severe injuries.

## Supplementary Information

Supplementary Material 1: Methods S1 “Plain Language Summary of Methods of Synthesis”, Table S1 “Risk of Bias”, Table S2 “Translational Effect Sizes”, Figure S1 “PRISMA-style flow diagram”, Figure S2 “Forest Plot of Comparative VAS Pain Scores (AUC) Across LAS Severity”, and Figure S3 “Forest Plot of Comparative VAS Pain Scores (AUC) Sensitivity Analysis on FAS-LOCF”.

## Data Availability

The datasets supporting the conclusions of this article are included within the article (and its additional files). Additional data are available within the original articles and supplementary files. Additional datasets generated during the TRAUMED study are not publicly available due to ongoing analysis. Subject to commercial considerations, they will subsequently be available from the trial sponsor for scientific purposes upon receipt of a reasonable request.
