# Supplementary material for: Tr14 gel compared to diclofenac gel after acute unilateral ankle sprain: an Individual Patient Data (IPD) meta-analysis of two multi-center trials
Source: BMC Musculoskelet Disord. 2026 May 1;27:372. doi: 10.1186/s12891-026-09802-0 (PMC13135254; doi:10.1186/s12891-026-09802-0)
Supplement: Supplementary file 1 — Supplementary Material 1: Methods S1 “Plain Language Summary of Methods of Synthesis”, Table S1 “Risk of Bias”, Table S2 “Translational Effect Sizes”, Figure S1 “PRISMA-style flow diagram”, Figure S2 “Forest Plot of Comparative VAS Pain Scores (AUC) Across LAS Severity”, and Figure S3 “Forest Plot of Comparative VAS Pain Scores (AUC) Sensitivity Analysis on FAS-LOCF”. [file 12891_2026_9802_MOESM1_ESM.docx]

**SUPPLEMENT**

**Supplementary Methods 1. Plain Language Summary of Methods of Synthesis**

In analysing the results of the two trials, a standard method was used to combine findings (fixed effect model, Hedges-Olkin), assuming both studies measured the same treatment effect. A funnel plot to assess publication bias was not included, as it is only considered meaningful when at least ten studies are available. The consistency of the results was evaluated using commonly accepted measures of variation. To assess percent changes from baseline adequately, a robust method called the Wei-Lachin test was applied. This approach is well-suited for small numbers of studies and does not rely on strict assumptions. It helps determine whether at least one study shows a true benefit from the treatment, with none showing harm. Finally, a test was performed to check for conflicting treatment effects between the two studies, to ensure that combining their results was appropriate.

**Supplementary Table 1. Risk of Bias**

An outline of the risk for bias^[[1]](#footnote-1)^ and support for the author’s judgement regarding study elements: ‘selection’, ‘performance’, ‘detection’, ‘attrition’, ‘reporting’ and ‘others’, within the TAASS (**A**) and the TRAUMED (**B**) studies.

**A.**

| **Bias** | **Authors' judgement** | **Support for judgement** |
| --- | --- | --- |
| Random sequence generation (selection bias) | Low risk | Random sequence generation described (computer-generated random numbers). |
| Allocation concealment (selection bias) | Low risk | Allocation was concealed using sealed, opaque, sequentially numbered envelopes prepared by an independent statistician. |
| Blinding of participants and personnel (performance bias) | Low risk | The trial was double-blind for both active gel groups. The drug preparations were packed in identical containers and the investigator in the study was blinded as well as the patient.  Blindings should only be broken in emergency situations for  reasons of subject’s safety. |
| Blinding of outcome assessment (detection bias) | Low risk | Outcome assessors were blinded to treatment allocation. |
| Incomplete outcome data (attrition bias) | Low risk | The dropout rate at the primary endpoint (Day 7) was below 1%, the dropout rate until the final visit (Day 42) was below 5%, with similar losses in both treatment groups. Reasons for missing data were described. |
| Selective reporting (reporting bias) | Low risk | No evidence of selective outcome reporting was found. Original Study Protocol and Clinical Trial Report including Individual Patient Data were available. |
| Other bias | Unclear risk | No other bias identified. |

**B.**

| **Bias** | **Authors' judgement** | **Support for judgement** |
| --- | --- | --- |
| Random sequence generation (selection bias) | Low risk | Random sequence generation described (computer-generated random numbers). |
| Allocation concealment (selection bias) | Low risk | Allocation was concealed using sealed, opaque, sequentially numbered envelopes prepared by an independent statistician. |
| Blinding of participants and personnel (performance bias) | Low risk | The trial was double-blind for both active gel groups. The drug preparations were packed in identical containers and the investigator in the study was blinded as well as the patient.  Blindings should only be broken in emergency situations for  reasons of subject’s safety. |
| Blinding of outcome assessment (detection bias) | Low risk | Outcome assessors were blinded to treatment allocation. |
| Incomplete outcome data (attrition bias) | Low risk | The dropout rate at the primary endpoints (Day 4 and Day 7) was below 1%, the dropout rate until the final visit (Day 14) was below 2%, with similar losses in both treatment groups. Reasons for missing data were described. |
| Selective reporting (reporting bias) | Low risk | No evidence of selective outcome reporting was found. Original Study Protocol and Clinical Trial Report including Individual Patient Data were available |
| Other bias | Unclear risk | No other bias identified. |

**Supplementary Table 2. Translational Effect Sizes**

Relevant benchmarks for well-known effect size measures are summarized in Table 2A. Table 2B, 2C and 2D show the translational effect sizes for VAS pain on passive movement (B), Time to 50% Pain Decrease (C), and FAAM (D). For each outcome, the original effect size measures as well as the derived translational effect size measures are provided.

1. **Relevant benchmarks for effect size measures**

| **Effect size measures and their benchmark values**  **for relevance of difference^+^** | | | | | | | |  |
| --- | --- | --- | --- | --- | --- | --- | --- | --- |
| **Magnitude^*^** | **SMD^§^** |  | **MW^*^** |  | **Odds Ratio^#^** |  | **NNT^$^** | |
| Small | 0.2 |  | 0.56 |  | 1.4 |  | 18 | |
| Medium | 0.5 |  | 0.64 |  | 2.3 |  | 7 | |
| Large | 0.8 |  | 0.71 |  | 3.9 |  | 5 | |
| ^+^With respect to distribution assumptions, results are to be interpreted as approximately ^*^Cohen Benchmarks for relevance of a difference^[[2]](#footnote-2),^^[[3]](#footnote-3),^^[[4]](#footnote-4),5^  ^§^Standardized Mean Difference (SMD); assumption of normal distribution^2,3,4^ | | | | | | | |  |
| ^*^Mann-Whitney effect size measure (MW); robust/assumption free^3,^^[[5]](#footnote-5)^  ^#^Odds Ratio (OR); derived from MW^3^; assumption of proportional odds^3^ | | | | | | | |  |
| ^$^Number needed to treat (NNT); derived from MW via RD_average_ ^3^ | | | | | | | |  |

The standardized mean difference (SMD) is a common effect size measure for continuous, normally distributed data and a general basis for important other distribution families. The well-known benchmark values of Cohen with respect to group differences - small, medium, large - can be used for assessing the relevance of difference. SMD can be translated into the corresponding Mann–Whitney (MW) effect size measure of stochastic superiority. MW is a robust alternative, needing no assumptions about a distribution family. From MW, benchmarks for parameters of other distribution families can be derived, such as odds ratio (OR), or number needed to treat (NNT).

1. **Pain on Passive Movement**

|  |  |  |  | **TRANSLATIONAL EFFECT SIZES** | | | | |
| --- | --- | --- | --- | --- | --- | --- | --- | --- |
| **Outcome** | **Statistical Method of Synthesis** | **Original Effect Size Measure** |  | **SMD^§^** | **MW** | | **OR** | **NNT** |
| PAIN ON PASSIVE MOVEMENT (VAS), AUC, ANCOVA, LOCF, PP |  |  |  |  |  | |  |  |
| AUC Day 4 | IV | Mean Difference | -6,90 | -0,15 | 0,54 | | 0,78 | 23 |
| AUC Day 7 | IV | Mean Difference | -26,30 | -0,31 | 0,59 | | 0,59 | 12 |
| AUC Day 14 | IV | Mean Difference | -63,60 | -0,37 | 0,60 | | 0,53 | 10 |
| PAIN ON PASSIVE MOVEMENT (VAS), %-CHANGES FROM BASELINE, LOCF, PP |  |  |  |  |  | |  |  |
| %-Change, Day 4 | WL | Mann-Whitney | 0,60 | -0,35 | 0,60 | | 0,56 | 10 |
| %-Change, Day 7 | WL | Mann-Whitney | 0,59 | -0,32 | 0,59 | | 0,58 | 11 |
| %-Change, Day 14 | WL | Mann-Whitney | 0,56 | -0,21 | 0,56 | | 0,70 | 17 |
| PAIN ON PASSIVE MOVEMENT (VAS), AUC, ANCOVA, LOCF, PP, GRADE SUBGROUPS |  |  |  |  |  | |  |  |
| AUC, Day 4 | IV | Mean Difference | -7,12 | -0,15 | 0,54 | | 0,77 | 23 |
| AUC, Day 7 | IV | Mean Difference | -24,61 | -0,30 | 0,58 | | 0,60 | 12 |
| AUC, Day 14 | IV | Mean Difference | -60,35 | -0,37 | 0,60 | | 0,53 | 10 |
| PAIN ON PASSIVE MOVEMENT (VAS), AUC, ANCOVA, LOCF, FAS |  |  |  |  |  | |  |  |
| AUC, Day 4 | IV | Mean Difference | -6,34 | -0,13 | 0,54 | | 0,80 | 27 |
| AUC, Day 7 | IV | Mean Difference | -24,33 | -0,28 | 0,58 | | 0,62 | 13 |
| AUC, Day 14 | IV | Mean Difference | -58,26 | -0,33 | 0,59 | | 0,57 | 11 |
| ^+^With respect to distribution assumptions, results are to be interpreted as approximately  ^§^As a rule of thumb, 0.2 SD represents a small difference, 0.5 a moderate and 0.8 a large | | | | | |  |  |  |

1. **Time to 50% Pain Decrease**

|  |  |  |  | **TRANSLATIONAL EFFECT SIZES** | | | |
| --- | --- | --- | --- | --- | --- | --- | --- |
| **Outcome** | **Statistical Method of Synthesis** | **Original Effect Size Measure** |  | **SMD^§^** | **MW** | **OR** | **NNT** |
| Time to 50% Pain Decrease, Days, ANCOVA (PP) |  |  |  |  |  |  |  |
| Absolute TIme (Days) | IV | Mean Difference | -1,58 | -0,40 | 0,61 | 0,51 | 9 |

^+^With respect to distribution assumptions, results are to be interpreted as approximately

^§^As a rule of thumb, 0.2 SD represents a small difference, 0.5 a moderate and 0.8 a large

1. **FAAM ADL (Activity of Daily Living Questionnaire)**

|  |  |  |  | **TRANSLATIONAL EFFECT SIZES** | | | | |
| --- | --- | --- | --- | --- | --- | --- | --- | --- |
| **Outcome** | **Statistical Method of Synthesis** | **Original Effect Size Measure** |  | **SMD** | **MW** | | **OR** | **NNT** |
| FAAM ADL, ANCOVA, LOCF, PP |  |  |  |  |  | |  |  |
| Absolute, Day 4 | IV | Mean Difference | -2,31 | -0,16 | 0,55 | | 0,76 | 22 |
| Absolute, Day 7 | IV | Mean Difference | -3,43 | -0,22 | 0,56 | | 0,68 | 16 |
| Absolute, Day 14 | IV | Mean Difference | -2,01 | -0,16 | 0,55 | | 0,76 | 22 |
| FAAM ADL, %-CHANGES FROM BASELINE, LOCF, PP |  |  |  |  |  | |  |  |
| %-Change, Day 4 | WL | Mann-Whitney | 0,55 | -0,18 | 0,55 | | 0,74 | 20 |
| %-Change, Day 7 | WL | Mann-Whitney | 0,59 | -0,32 | 0,59 | | 0,58 | 11 |
| %-Change, Day 14 | WL | Mann-Whitney | 0,55 | -0,19 | 0,55 | | 0,72 | 18 |
| Time to 50% Pain Decrease, Days, ANCOVA (PP) |  |  |  |  |  | |  |  |
| Absolute TIme (Days) | IV | Mean Difference | -1,58 | -0,40 | 0,61 | | 0,51 | 9 |
| ^+^With respect to distribution assumptions, results are to be interpreted as approximately  ^§^As a rule of thumb, 0.2 SD represents a small difference, 0.5 a moderate and 0.8 a large | | | | | |  |  |  |

**Abbreviations in Table B, C, D**

AUC = Area Under the Curve

ANOVA = Analysis of Covariance

VAS = Visual Analogue Scale

FAAM = Foot and Ankle Ability Measure - Activities of Daily Living (21-item self-report questionnaire subscale)

PP Per-Protocol

LOCF = Last Value Carried Forward

FAS = Full Analysis Set

IV = Inverse Variance Model

WL = Wei-Lachin test of stochastic ordering (one-dimensional test)

SMD = Standardized Mean Difference

MW = Mann-Whitney Effect Size Measure

OR = Odds Ratio

NNT = Number needed to treat

**SUPPLEMENTARY FIGURES**

**Supplementary Figure 1. PRISMA-style flow diagram.**^[[6]](#endnote-1)^

A flow chart detailing the identification and screening of eligible trials with available IPD for this focused IPD meta-analysis

**
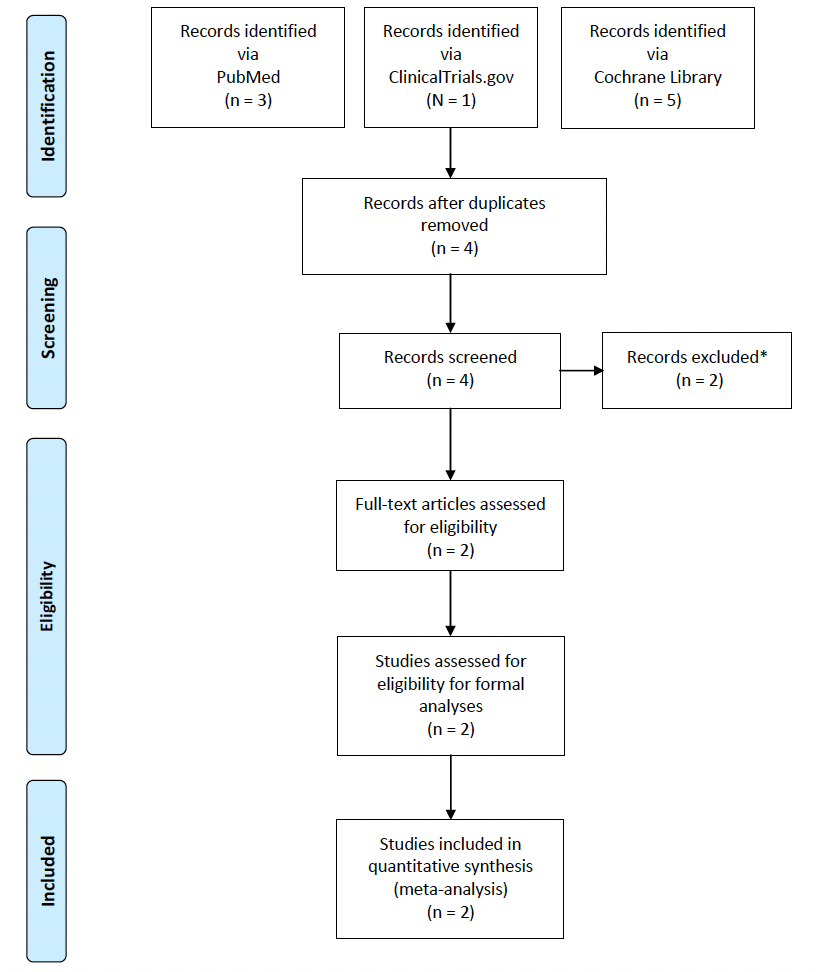
**

^*^Exclusions: (1) observational cohort study, (2) Tr14^[[7]](#footnote-6)^ ointment vs. placebo

*From:*  Moher D, Liberati A, Tetzlaff J, Altman DG, The PRISMA Group (2009).
*P*referred *R*eporting *I*tems for *S*ystematic Reviews and *M*eta-*A*nalyses: The PRISMA Statement.
PLoS Med 6(7): e1000097. doi:10.1371/journal.pmed1000097

**Supplementary Figure 2. Forest Plot of Comparative VAS Pain Scores (AUC) Across LAS Severity.**

Following exposure to Experimental (Tr14 gel), or Control (diclofenac gel), VAS pain scores were measured on Days 4, 7, and 14, and were analysed via AUC ANCOVA (applied to IPD within Grade Subgroups with PP-LOCF). Effect sizes are expressed as ‘Mean Difference’ with associated confidence intervals for Patients with a Grade 1 LAS in TAASS (small green square), TRAUMED (large green square), and Subtotal (black rhombus), and for Patients with a Grade>1 LAS in TAASS (small green square), TRAUMED (small green square), and Subtotal (black rhombus), with additional Total ‘Mean Difference’ (lower black rhombus). LAS Grade 1 = mild impairment, LAS Grade >1 = moderate to severe impairment.

**Day 4**

**
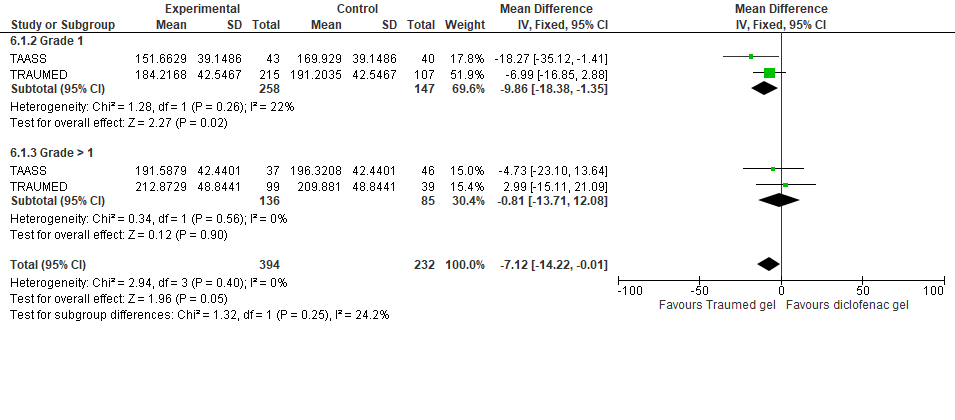
**

**Day 7**

**
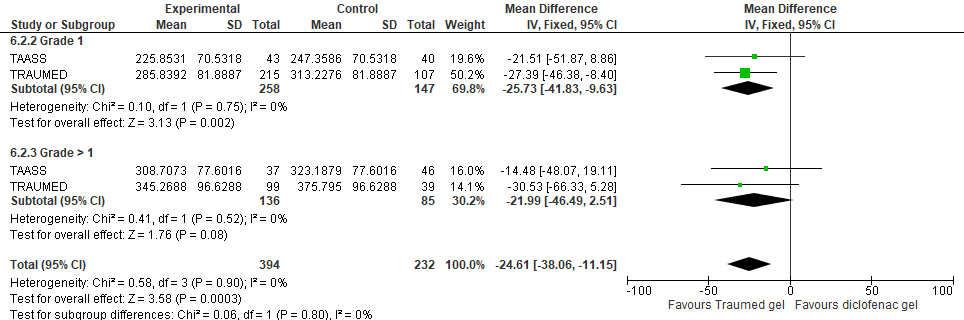
**

**Day 14**

**
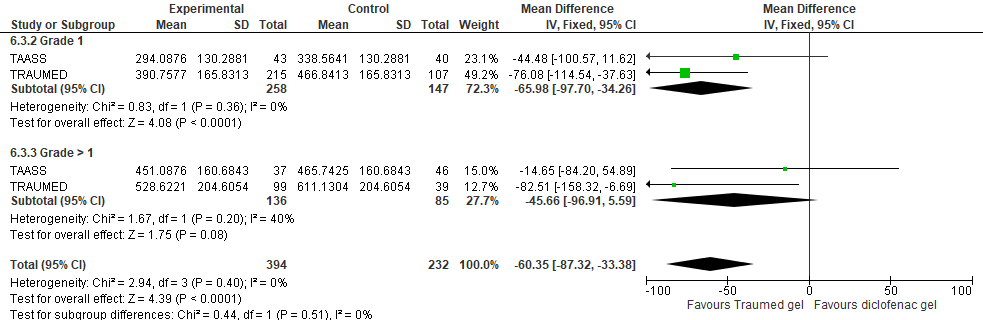
**

**Abbreviations:** Traumed gel (Tr14 gel), LAS (Lateral Ankle Sprain), VAS (Visual Analogue Scale), ANCOVA (Analysis of Covariance), IPD (Individual Patient Data), PP (Per-Protocol), LOCF (Last Value Carried Forward), IV (Inverse Variance), Fixed (Fixed Effect Model), CI (Confidence Interval), SD (Standard Deviation).

1. Moher D, Liberati A, Tetzlaff J, Altman DG, The PRISMA Group (2009). *P*referred *R*eporting *I*tems for *S*ystematic Reviews and *M*eta-*A*nalyses: The PRISMA Statement. PLoS Med 6(7): e1000097. doi:10.1371/journal.pmed1000097. [↑](#footnote-ref-1)
2. Cohen J. *Statistical Power Analysis in the Behavioral Sciences*. 2nd edition ed. Hillsdale (NJ): Lawrence Erlbaum Associates, Inc.; 1988. [↑](#footnote-ref-2)
3. Rahlfs V and Zimmermann H. Effect size measures and their benchmark values for quantifying benefit or risk of medicinal products. *Biom J* 2019; 61: 973-982. 20190228. DOI: 10.1002/bimj.201800107. [↑](#footnote-ref-3)
4. Schünemann HJ, Vist GE, Higgins JPT, Santesso N, Deeks JJ, Glasziou P, Akl EA, Guyatt GH. Chapter 15.5: Interpreting results from continuous outcomes (including standardized mean differences) [last updated August 2023]. In: Higgins JPT, Thomas J, Chandler J, Cumpston M, Li T, Page MJ, Welch VA (editors). *Cochrane Handbook for Systematic Reviews of Interventions* version 6.5. Cochrane, 2024. [↑](#footnote-ref-4)
5. Colditz GA, Miller JN and Mosteller F. Measuring gain in the evaluation of medical technology. The probability of a better outcome. *Int J Technol Assess Health Care* 1988; 4: 637-642. DOI: 10.1017/s0266462300007728. [↑](#footnote-ref-5)
6. [↑](#endnote-ref-1)
7. Tr14 gel (Traumeel®, Traumed, Heel GmbH, Baden-Baden) is a combination of 14 ingredients, mainly plant phenol extracts. [↑](#footnote-ref-6)
